# Multiple Patterns of *FHIT* Gene Homozygous Deletion in Egyptian Breast Cancer Patients

**DOI:** 10.4061/2011/325947

**Published:** 2011-10-19

**Authors:** Heba M. S. Ismail, Amina M. Medhat, Amr M. Karim, Nadia I. Zakhary

**Affiliations:** ^1^Cancer Biology Department, National Cancer Institute, Cairo University, Cairo, Egypt; ^2^Biochemistry Department, Faculty of Science, Ain Shams University, Cairo, Egypt

## Abstract

Fragile histidine triad (*FHIT*) gene encodes a putative tumour suppressor protein. Loss of Fhit protein in cancer is attributed to different genetic alterations that affect the *FHIT* gene structure. In this study, we investigated the pattern of homozygous deletion that target the FHIT gene exons 3 to 9 genomic structure in Egyptian breast cancer patients. We have found that 65% (40 out of 62) of the cases exhibited homozygous deletion in at least one FHIT exon. The incidence of homozygous deletion was not associated with patients' clinicopathological parameters including patients' age, tumour grade, tumour type, and lymph node involvement. Using correlation analysis, we have observed a strong correlation between homozygous deletions of exon 3 and exon 4 (*P* < 0.0001). Deletions in exon 5 were positively correlated with deletions in exon 7 (*P* < 0.0001), Exon 8 (*P* < 0.027), and exon 9 (*P* = 0.04). Additionally, a strong correlation was observed between exons 8 and exon 9 (*P* < 0.0001).We conclude that *FHIT* gene exons are homozygously deleted at high frequency in Egyptian women population diagnosed with breast cancer. Three different patterns of homozygous deletion were observed in this population indicating different mechanisms of targeting *FHIT* gene genomic structure.

## 1. Introduction

Fragile histidine triad (*FHIT*) gene located at chromosome 3p14.2 is a putative tumour suppressor gene involved in the pathogenesis of breast cancer. Both genetic and epigenetic alterations in *FHIT* have been implicated in breast carcinoma [1–3]. Consistent with its proposed function as a tumour suppressor, homozygous genomic deletions within the *FHIT *gene have been observed in a large number of human cancers and cancer cell lines. *FHIT* is a member of histidine triad (HIT) proteins, which represent a small family of nucleotide-binding and hydrolyzing proteins. HIT proteins received attention of cancer biologists because of their downregulated expression in multiple human malignancies [4]. *FHIT* gene deletion or loss of transcription has been reported in head and neck [5], gastrointestinal [6, 7], cervical [8], lung [9] breast, [10, 11], kidney [12], and hematopoietic tumours [13]. Chromosomal region 3p14.2 is a frequent target for genetic and cytogenetic alterations in a wide range of solid tumors [14], leading to the search for a tumour suppressor gene in this region.

 The function of *FHIT* is directly linked to intracellular signalling and the DNA damage response [15]. *FHIT* is frequently involved in biallelic loss and other chromosome abnormalities in tumours [16, 17]. *FHIT* deletions, abnormal transcripts, promoter hypermethylation, and associated loss of expression are common in human malignancies. In cancer cells, these events are often associated with deletions directly within the FRA3B region, centering on exon 5 of *FHIT*. A high frequency of heterozygous and homozygous deletions with breakpoints within the fragile site region of *FHIT* has been detected in large number of cancers and cancer cell lines [5, 18–21]. Corbin et al. [22] showed multiple, variable deletions in FRA3B, even in cells derived from the same tumour, suggesting ongoing instability in the region. 

The *FHIT* locus contains the most fragile site in the human genome; FRA3B, the papilloma virus integration site, and a familial-kidney-cancer-associated breakpoint *t*(3;8) (p14.2;q24) [16, 35]. *FHIT* gene has 10 small exons extend over ~2 Mb DNA that make up the 1.1 kb cDNA [23]. Exons 5 to 9 are the coding exons that encode a small protein of 16.8 KD mass. Fhit protein is a typical dinucleoside 5′,5′′′-P1, P3-triphosphate (Ap3A) hydrolase which is highly homologous to Ap4A (diadenosine tetraphosphate) hydrolase from *Schizosaccharomyces pombe*. The Fhit protein exerts its oncosuppressor activity through induction of an apoptotic mechanism that seems to be Fas-associated death domain (FADD) dependent, caspase-8 mediated, and independent from mitochondrial amplification [24]. Loss of Fhit protein in cancer is attributed to different genetic alterations that affect *FHIT* gene structure. In tumour-derived cell lines, homozygous deletions that result from the loss of genomic regions containing or surrounding the relevant *FHIT *exons lead to numerous abnormal transcripts that included the absence of various regions between exon 4 and 9, while the mRNAs of the corresponding normal tissues did not exhibit these alterations [16]. None of the truncated transcripts can encode a fully functional protein. In addition to homozygous deletion, *FHIT* gene structure is also subjected to loss of heterozygosity (LOH) and promoter hypermethylation [25]. The significant association of *FHIT* mutation and hypermethylation leads to the complete inactivation of *FHIT* gene in patients with breast cancer. Silencing of the *FHIT* gene by promoter hypermethylation occurs in breast carcinomas, especially those with the significant amount of mutations in its genomic structure [26]. *FHIT* gene methylation was also reported in serum of sporadic breast cancer in a CpG island methylator phenotype (CIMP) screening [27]. 

Despite the extensive analysis of *FHIT* gene in cancer cells, the studies on primary malignant tissues are limited. Here we aimed to understand the genetic alterations that affect *FHIT* gene in an Egyptian population of primary breast cancer patients. Recently, we have reported loss of heterozygosity (LOH) in* FHIT* gene locus and its flanking region on chromosome 3p in Egyptian breast cancer patients [28]. To extend the analysis of *FHIT* gene locus in this population, we investigated the incidence of homozygous deletion that targets the *FHIT* gene exons and the correlation between observed deletions. Furthermore, we investigated the association between these deletions and the patients' clinicopathological data. 

## 2. Materials and Methods

### 2.1. Patients

Paired normal and tumour tissues were obtained from 62 patients diagnosed with breast carcinomas prior to therapy at the National Cancer Institute (Cairo, Egypt). Matched normal tissue samples were obtained from the tumour safety margin. The study was approved by the review board of the institution. The specimens collected and used in the study were obtained under each patient's consent along with approval. The patients were staged according to the TNM Staging System [29]. The patients' clinic-pathological parameters are summarised in Table 1.

### 2.2. DNA Extraction

DNA extraction from the tumour and safety margin tissues obtained from patients was performed using QIAamp DNA Minikit method (Qiagen, Germany) as described in the manufacturer's protocol. This method depends on degradation of the proteins and cell membranes using proteinase K enzyme, precipitation of DNA by ethanol (96–100%) followed by purification of DNA using QIAamp spin columns. The retained DNA is finally eluted from the membrane using TE buffer.

### 2.3. Homozygous Deletion Analysis

Patients DNAs were examined for homozygous deletion in *FHIT* exons. In brief, PCRs were performed with 8 different primers corresponding to exons 3 to 9, and *β*-actin was used as a control. Exon-specific primers used in this study are listed in Table 2. PCR amplification was performed in a 12.5 *μ*L reaction volume using 1× PCR buffer (Invitrogen), 1.5 mM MgCl_2_ (Invitrogen), 0.2 mM of each dNTP, 20 ng of each primer, 0.5 unit Taq polymerase (Invitrogen), and 100 ng template DNA. The reaction mixture was amplified using Perkin Elemer thermal cycler. Cycling conditions were 94°C for 2 min followed by 30 cycles at 94°C for 30 s, 57°C for 30 s, and a final extension step at 72°C for 7 min. PCR products were then electrophoresed onto 2% agarose gel stained with ethidium bromide and visualized under UV transilluminator. Homozygous deletions were evaluated by comparing bands of exons in tumour samples with their matched normals. Exon bands that showed significant reduction compared to normal were considered positive for HD.

### 2.4. Statistical Analysis

The results were analysed using GraphPad prism computer system (GraphPad software, San Diego, CA). Pearson correlation analysis was used to test the correlation between exons in the context of homozygous deletion. Chi-square and Fisher exact tests were used to test the association between or HD deletions with each of patients' clinicopathological parameters. The association was considered significant when *P* ≤ 0.05.

## 3. Results

### 3.1. FHIT Exons Are Subjected to Homozygous Deletion in Breast Cancer Patients

*FHIT* gene has 10 small exons that make up the 1.1-kb cDNA [23]. Exons 3, 4, and 5 are present in close proximity to the familial kidney-tumour-associated *t* (3; 8) translocation break, including the HPV16 integration site identified in cervical carcinoma, and the fragile sites in intron 5. Out of 10 exons, the first four exons and the last exon are noncoding exons; therefore, exon 5 is the first coding exon of the *FHIT* gene which has the initial methionine codon of the *FHIT *open reading frame (ORF), that extends to exon 9 and encodes a small protein of 16.8 KD mass. while exon 8 encodes the histidine triad motif [16]. 

To investigate the incidence of homozygous deletion in *FHIT* gene exons in Egyptian patients diagnosed with breast cancer, we performed exon-specific PCRs for coding exons 5 to 9. Additionally, we investigated noncoding exons 3 and 4.

**Figure 1:**
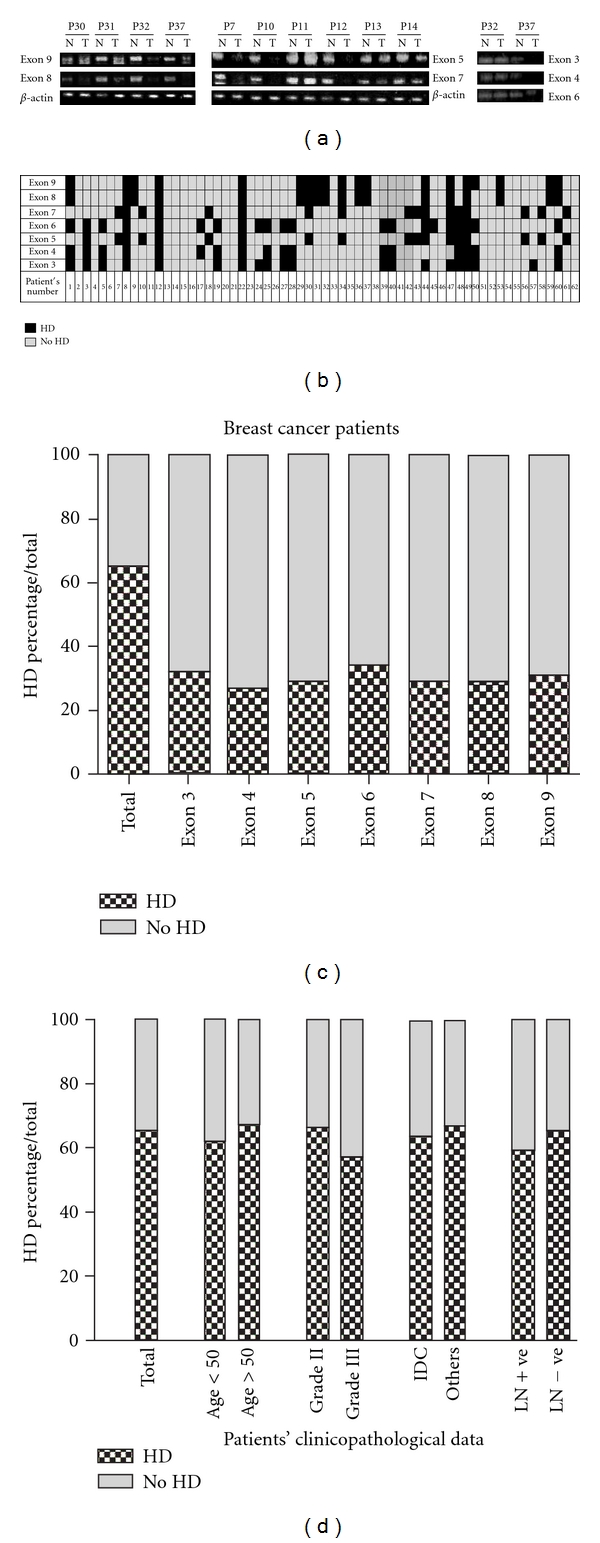
*FHIT* gene exons are subjected to homozygous deletions in Egyptian neoplastic and preneoplastic breast tissues. DNA was extracted from breast cancer and matched normal tissue samples then subjected to PCR analysis of FHIT exons. (a) Amplification of exons 3, 4, 5, 6, 7, 8, and 9. *β*-actin was used as a control for equal amount of DNA used in the amplification. Intensity of amplified band in tumour (T) samples was compared with its relative one in normal sample (N). Samples that showed significant reduction or loss of the amplified exons were considered positive for exon homozygous deletion (HD). (b) Cumulative data of HD in individual patients. (C) Incidence of HD of *FHIT* exons in breast cancer patients. (c) Incidence of HD of *FHIT* exons in breast cancer patients. (d) Association between HD of* FHIT* exons and breast cancer patients' clinicopathological parameters.

A total of 62 breast cancer patients were investigated for homozygous deletion (HD) in *FHIT* exons. Tumour DNA was compared to its matching normal DNA samples isolated from the tumour safety margin. We found that 65% of the cases (40 out of 62) showed HD in at least one of the seven examined exons. Percentage of HD in the examined exons is as follows: exon 3: 32.3% (20/62), exon 4: 27.4% (17/62), exon 5: 29% (18/62), exon 6: 34% (21/62), exon 7: 29% (18/62), exon 8: 29% (18/62), exon 9: 30.6% (19/62). We have observed that the samples which exhibited HD in exon 8 were also positive for HD in exon 9. Furthermore, 95% of samples that showed HD in exon 4 were also positive for HD in exon 3. Moreover, 29% of the total samples exhibited deletions in the exons cluster from 5 to 7.  Figure 1(a) shows representative samples that exhibited HD in the investigated exons. Cases no. 7, 10, and 12 showed HD in exons 5 and 7 in tumour samples compared to their normal counterparts, while cases 13 and 14 showed intact exons in normal and tumour samples. Cases no. 30, 31, 32, and 37 exhibited HD in exons 8 and 9 in tumour samples compared with their normal counterparts. Case no. 37 exhibited HD in exons 3 and 4, while exon 6 is intact compared to its matched normal. Case no. 32 showed intact exons 3, 4 and 6. Figure 1(b) shows the incidence of HD in *FHIT* exons in individual breast cancer patients. A summary of HD percentages in *FHIT* exons is shown in Figure 1(c).

From these data, we conclude that homozygous deletion or HD of *FHIT* gene exons occurs at high frequency in Egyptian breast cancer patients. The high deletion rate indicates that *FHIT* gene is subjected to intragenic breaks and rearrangements and homozygous deletion of *FHIT* gene is an important event in breast carcinogenesis.

### 3.2. Association between Homozygous Deletions of FHIT Exons and Patients' Clinicopathological Parameters

*FHIT* exons deletion in breast cancer samples were compared with patients' clinicopathological parameters including tumour type, tumour grade, patient's age at diagnosis, and lymph node status. Results were tested for statistical significance difference using Chi-square and fisher exact test. 

In breast cancer set, no significance association was observed between HD in *FHIT* exons and patients clinicopathological data. Patients younger than 50 years exhibited HD in 18 out of 29 cases (62%), while 18 out of 27 cases (67%) showed deletions in patients of ages above 50 years old (*P* = 0.7849). Among the 50 samples diagnosed with invasive ductal carcinoma type, 32 samples (64%) showed HD, whereas 8 samples out of 12 (67%) of 12 patients diagnosed with invasive lobular carcinoma and other types showed HD (*P* = 0.8624). Samples' tumour grades varied from grade II to III. Out of 35 cases with grade II, 23 (66%) showed HD while, and 8 out of 14 cases (57%) of grade III showed HD (*P* = 0.57). With lymph node status, 10 out of 17 (59%) showed HD in lymph node negative set, while in lymph node positive cases 24 out of 37 samples (65%) showed HD (*P* = 0.6694). 

Taken those data together, we conclude that FHIT gene could play an important role in breast carcinogenesis, and its alterations are not restricted with certain features of the disease. This means that the allelic loss in FHIT gene structure can occur in breast cancer patients regardless of patient's age, tumour grade, tumour type, and lymph node involvement.

### 3.3. Classification of FHIT Gene Exons Homozygous Deletion in Breast Cancer Samples

**Figure 2:**
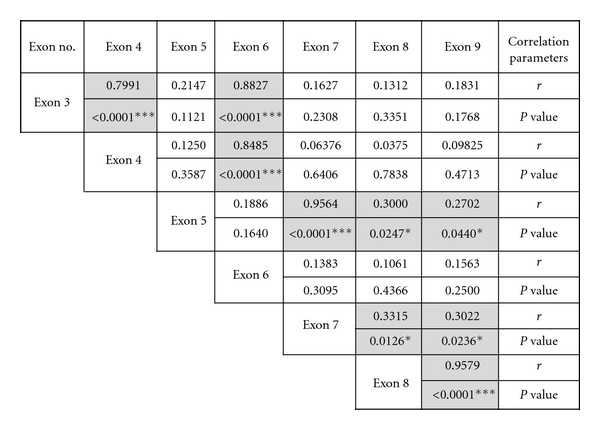
Correlation analysis between *FHIT* exons homozygous deletions. Correlation analysis was done using Pearson and Spearman correlation tests; “*r*” is correlation coefficient. Highlighted values represent significant positive correlation between the two variables. *P < 0.05, ***P < 0.0001.

Having identified the incidence of HD at each exon in individual patients, we then investigated the pattern of detected deletions. To achieve that, we performed statistical analysis of correlation between all exons and the incidence of HD using Pearson and spearman correlation tests. The spearman correlation coefficient and *p* values among investigated exons are shown in Figure 2. 

Interestingly, a strong positive correlation was detected between the HD incidence in the non-coding exons 3 and 4 (*r* = 0.7991,  *P* < 0.0001), while there was no correlation between HD incidence in any of those exons and exons 5, 7, 8, and 9. This can be considered as the first deletion cluster. The second deletion cluster involved exons 5, 6, and 7 a strong positive correlation was detected between exons 5 and 7 (*r* = 0.9564, *P* < 0.0001) but not 6. Exon 5 deletions were also positively correlated with deletions in exon 8 and exon 9 (*P* = 0.02, *P* = 0.04). The third exon deletion cluster includes exons 8 and 9 which showed a significant positive correlation in HD incidence (*r* = 0.9579, *P* < 0.0001). 

From those data we can classify the observed deletions of *FHIT* exon into three different classes: class I includes exons 3 and 4, class 2 includes exons 5 and 7, and class III includes the distal exons 8 and 9. In some samples we can see deletions of exons from 5 to 9. Taken all those observations together we conclude that *FHIT* gene exons are targeted by three different mechanisms that cause homozygous deletions in the exon clusters.

## 4. Discussion

Consistent with its proposed function as a tumour suppressor, homozygous genomic deletions within the *FHIT* gene have been observed in a large number of human cancers and cancer cell lines. *FHIT *is one of the several tumour-suppressor genes on chromosome 3, when working normally, keeping any potentially cancerous cells from growing out of control. However, when mutations or alterations eliminate one or both copies of the *FHIT *gene, the “brakes” that control cell growth are released, allowing potentially cancerous lesions to become malignant.

*FHIT* gene was cloned by Ohta et al. 1996 [16]. Although the hypothesis that *FHIT* is a tumour suppressor gene was initially met with some scepticism, data in support of this function have been heavily accumulated. *FHIT* knockout mice developed spontaneous tumours and are more susceptible to cancer than wild-type mice. Additionally, Fhit suppresses tumourigenicity in cancer cell lines which implies conclusively that *FHIT* is a bona fide tumour suppressor gene [30]. The presence of the most common fragile site FRA3B within *FHIT* suggests that the fragility of this gene makes it susceptible to rearrangements induced by a variety of environmental carcinogens and cancer susceptibility reviewed in [31]. 

In the present study, we aimed to investigate the frequency of exon deletions that affect the *FHIT* gene in a series of primary breast cancer tumours in Egyptian population. We then investigated the correlation between all exons deletions. Furthermore, we sought after the association of these alterations with patients' clinicopathological data. 

Here, we found that 65% of the breast cancer patients investigated showed homozygous deletions (HDs) in at least one *FHIT* exon (40 out of 62). Deletions percentages in the coding exons 5–9 were as follows: exon 5: 29% (18/62), exon 6: 34% (21/62), exon 7: 29% (18/62), exon 8: 29% (18/62), exon 9: 30.6% (19/62). Deletions in the noncoding exons were observed as follows; exon 3: 32.3% (20/62), exon 4: 27.4% (17/62). This means that coding exons were deleted in 63% of the investigated samples while noncoding exons were deleted in about 32% of the cases. The incidence of HD in our samples was relatively higher than what is reported in previous studies. This can be attributed to higher frequency of rearrangements and breaks in the *FHIT* locus in this population. 

We have noticed that 58% of HD detected in *FHIT* gene exons are discontinuous deletions. Simply, deletions in these samples exhibited a specific pattern, for example, in some cases exon 4, 6, 8, and 9 were deleted, while exons 5 and 7 were intact. Discontinuous homozygous deletions in *FHIT* gene were reported in previous studies. Various authors observed this discontinuity of HD in some breast cell lines [1, 32, 33] which is consistent with our results in primary breast cancer. Ohta et al. (1996) and Druck et al. (1997) observed that homozygous deletions can occur in three or four discontinuous segments in FRA3B, and frequently involve loss of both copies of specific* FHIT* exons [16, 34]. Overall, this discontinuity can be explained by the possibility that the mechanism of breakage of the FRA3B, frequently allows multiple gaps to form on the same chromosome 3p, which when repaired leave multiple deletions simultaneously. Most of the deletions described here are predicted to lead to the truncation of Fhit protein [35]. It is not necessary that deletions have to occur in all the *FHIT* exons, but deletions in any of these exons will encode a truncated protein which might be functionless [20]. In the study of Campiglio et al. 1999, alterations in *FHIT* transcripts were detected in 31% of the patients but the reduction or absence of *FHIT* protein occurred in 69% of the breast carcinoma samples [2].

Analysis of *FHIT* gene in a series of human primary breast cancer revealed an allelic loss in 25% [1] and abnormal transcripts in approximately 30% of the cases [36]. *FHIT* homozygous deletions in samples with 3p14.2 aberrations were also found in the benign breast lesions of two women with familial tendency to breast cancer [37]. Another study of normal breast epithelium, breast preneoplastic lesions, and invasive tumors reported the loss of heterozygosity of the *FHIT* locus in two patients with intraductal hyperplasia [38]. We have studied a series of benign breast lesions and observed a high frequency rate of homozygous deletions in the noncoding exons (data not shown). 

Campiglio et al. [2] analyzed 29 cases of primary breast tumours for normal and abnormal *FHIT* transcripts and for the level of expression of *FHIT* protein in the normal breast epithelia and breast epithelia tumour of the same patient. Downmodulation or absence of *FHIT* protein was also evaluated in a series of 156 consecutive patients with primary breast carcinomas. In both groups, *FHIT* protein levels were reduced or absent in almost 70% of the breast cancer samples, whereas aberrant *FHIT* transcripts were detected in only 31% of the samples. Moreover, down-regulation of *FHIT* protein expression is associated with highly proliferative and large tumours. Yang et al. suggested that *FHIT* gene therapy may potentially be a clinically useful tool for the treatment of breast cancer [39]. 

Carefully observing the deletion patterns of *FHIT* exons in our samples, we identified three different deletion clusters based on correlation analysis in this population. First cluster includes the noncoding exons 3 and 4. We have also observed that the cluster containing exons 3, 4 was deleted in 29% of the breast cancer patients. A positive correlation was detected between those two exons (*r* = 0.7991, *P* < 0.0001) this indicates that this cluster is highly subjected to deletions in this population. It was reported that this exon cluster is present in close proximity to the familial-kidney-tumour-associated *t* (3;8) translocation break, the cluster of fragile sites identified by aphidicolin-induced chromosome breaks in human-hamster hybrid cells. This also includes the HPV16 integration site identified in cervical carcinoma and the fragile sites in intron 5 in aphidicolin-treated hybrid cells [16]. The deletions we observe could be due to breaks or viral integration in that region. Investigation of HPV16 infection incidence in this primary tumour series could explain why this region is subjected to deletions. The second deletion cluster includes exons 5 and 7 which are deleted in 27% of all samples. We observed a strong positive correlation was observed between deletions in exon 5 and exon 7 (*r* = 0.9564, *P* < 0.0001) but not exon 6. Deletions in exon 5 are expected to affect the gene transcription since it has the initial methionine codon of the *FHIT *open reading frame (ORF), resulting in the loss of the intact ORF [16]. Deletions in this region could be attributed to the rearrangements and breaks events that happen due to the presence of the fragile site FRA3B. The third deletion cluster includes the distal coding exon 8 and 9 which showed HD in 29% of the total breast cancer samples investigated. Interestingly, all samples that showed deletions in exon 8 were also positive for exon 9 deletions (*r* = 0.9579, *P* < 0.0001). The mechanism of deletion involving both exons together indicates their common importance especially exon 8 which flanks the histidine triad motif. Since exon 8 contains the histidine triad domain, it is suggested that this exon deletions could result in a nonfunctional protein. As a target of deletion, exon 8 could contain an essential function that is lost in carcinogenesis process. The presence of those three distinguishable deletion patterns may reflect the diverse mechanisms that could target the *FHIT* gene exons in tumourigenesis. 

We also have investigated the association between patients' clinic pathological parameters and the HD incidence in *FHIT* gene exons. No significant association was between HD in breast cancer patients and their clinicopathological features as age, lymph node involvement, tumour type, and tumour grade. This indicates that the deletions if *FHIT* exons are not restricted to subcategories of those parameters.

Overall, we show here that *FHIT* genomic structure is subjected to extensive allelic alterations represented by exon homozygous deletion in a series of Egyptian primary breast cancer. The presence of homozygous deletion of *FHIT* exons in breast cancer samples implicates that alteration in *FHIT* gene is an important event in breast pathogenenesis in this population. There was no association between observed deletions and the patients clinicopathological parameters. Additionally, we have identified three different patterns of homozygous deletion which could reflect different mechanisms that target exon deletion in *FHIT* structure.* FHIT* gene is a crucial tumour suppressor gene whose inactivation may derive clonal expansion of preneoplastic and neoplastic cells in the breast. The present findings highlight the *FHIT* gene as an interesting target for extensive analysis in Egyptian breast cancer patients.

## Figures and Tables

**Table 1:** Patients clinicopathological features.

Sample no.	Age	Tumour type	Grade (G)	Lymph node status
1	77	IDC	G2	0/5
2	47	IDC	G2	0/9
3	52	IDC	G2	0/12
4	62	IDC	G2	0/8
5	40	ILC	NI	18/18
6	NI	Angiosarcoma	G3	3/19
7	52	IDC	G2	6/6
8	50	ILC	NI	7/9
9	42	IDC	G2	3/12
10	35	IDC	G2	2/13
11	40	IDC	G2	5/14
12	40	ILC	NI	3/14
13	55	IDC	G2	NI
14	38	IDC	G2	5/8
15	50	IDC	G2	17/19
16	42	IDC	G2	0/17
17	NI	IDC	G3	NI
18	50	ILC	NI	0/13
19	58	IDC	G3	14/15
20	41	IDC	G2	4/11
21	46	IDC	G3	0/19
22	42	IDC	G2	2/13
23	50	ILC	NI	7/7
24	44	IDC	G3	0/12
25	55	IDC	G2	5/12
26	41	IDC	G3	0/24
27	34	IDC	G2	9/12
28	53	IDC	G2	0/9
29	75	IDC	G2	0/7
30	55	Comedocarcinoma	NI	NI
31	NI	IDC	G2	NI
32	60	IDC	G2	0/16
33	50	IDC	G3	11/13
34	42	IDC	G3	6/22
35	70	IDC	G2	3/17
36	45	Adenocarcinoma	NI	1/14
37	61	IDC	G2	5/18
38	NI	IDC	NI	0/10
39	58	IDC	G3	0/8
40	52	IDC	G2	1/7
41	42	IDC	G2	1/15
42	55	IDC	G2	1/13
43	35	IDC	G2	15/15
44	45	IDC	G2	0/12
45	40	IDC	G2	11/18
46	55	IDC	G2	6/22
47	40	IDC	G3	11/20
48	35	IDC	G3	6/10
49	55	IDC	G2	4/15
50	34	IDC	G2	4/11
51	50	IDC	G3	1/13
52	65	IDC	G2	5/8
53	62	IDC	G2	4/15
54	42	IDC	G3	0/10
55	40	ILC	G1	NI
56	NI	IDC	NI	NI
57	50	Medullary carcinoma	NI	NI
58	35	IDC	G3	4/12
59	40	IDC	G3	6/22
60	73	Gynecomastia*	NI	NI
61	42	IDC*	G2	5/12
62	44	ILC	G1	NI

IDC: invasive ductal carcinoma, ILC: invasive lobular carcinoma, and NI: no information.

*Male patients.

**Table 2:** Sequence of primers used in this study.

Exon	Sequence	Expected size (BP)
Exon 3	3F: 5′ AGGGTGATACTAGCTGCTTT 3′	284
	3R: 5′ TGACTTTAGCCAGTGGCA 3′	

Exon 4	4F: 5′ TTGTACCTAGAGCCATCTGG 3	224
	4R: 5′ GGATACTCACAGCAGGTCAA 3′	

Exon 5	5F: 5′ TGAGGACATGTCGTTCAGATTT 3′	255
	5R: 5′ CTGGTGTCTCCGAAGTGGGAGGG 3′	

Exon 6	6F: 5′ ATGTTCTTGTGTGCCCGCTGCGGCCAGT 3′	147
	6R: 5′ CTGCATGGAAAAGGTGAGAGAGGTCCCATG 3′	

Exon 7	7F: 5′ TGGTCCCCATGAGAATACTATAAATTAACA 3′	305
	7R: 5′ TTACGGCTCTAACACTGAGGGTCTCTCTGA 3′	

Exon 8	8F: 5′ GAGTAATTGGGCTTCATGAGAGCATCACT 3′	225
	8R: 5′ AGGTTGATGTCATCCCACCGACAGT 3′	

Exon 9	9F: 5′ TTCTCCAAAGCTCCAGAAACATGACAAGGA 3′	119
	9R: 5′ GTCTTTACCTGTGTCACTGAAAGTAGACCC 3′	

*β*-actin	F: 5′ TCATCACCAATTGGCAATGAG 3′	147
	R: 5′ CACTGTGTTGGCGTACAGGT 3′	

## References

[1] Negrini M, Monaco C, Vorechovsky I (1996). The FHIT gene at 3p14.2 is abnormal in breast carcinomas. *Cancer Research*.

[2] Campiglio M, Pekarsky Y, Menard S, Tagliabue E, Pilotti S, Croce CM (1999). FHIT loss of function in human primary breast cancer correlates with advanced stage of the disease. *Cancer Research*.

[3] Iliopoulos D, Guler G, Han SY (2005). Fragile genes as biomarkers: epigenetic control of WWOX and FHIT in lung, breast and bladder cancer. *Oncogene*.

[4] Huber O, Weiske J (2008). *β*-catenin takes a HIT. *Cell Cycle*.

[5] Virgilio L, Shuster M, Gollin SM (1996). FHIT gene alterations in head and neck squamous cell carcinomas. *Proceedings of the National Academy of Sciences of the United States of America*.

[6] Baffa R, Veronese ML, Santoro R (1998). Loss of FHIT expression in gastric carcinoma. *Cancer Research*.

[7] Michael D, Beer DG, Wilke CW, Miller DE, Glover TW (1997). Frequent deletions of FHIT and FRA3B in Barrett’s metaplasia and esophageal adenocarcinomas. *Oncogene*.

[8] Hendricks DT, Taylor R, Reed M, Birrer MJ (1997). FHIT gene expression in human ovarian, endometrial, and cervical cancer cell lines. *Cancer Research*.

[9] Sozzi G, Veronese ML, Negrini M (1996). The FHIT gene at 3p14.2 is abnormal in lung cancer. *Cell*.

[10] Bianchi F, Tagliabue E, Ménard S, Campiglio M (2007). Fhit expression protects against HER2-driven breast tumor development: unraveling the molecular interconnections. *Cell Cycle*.

[11] Pandis N, Bardi G, Mitelman F, Heim S (1997). Deletion of the short arm of chromosome 3 in breast tumors. *Genes Chromosomes and Cancer*.

[12] Lee SH (2006). Differential gene expression in nickel(II)-treated normal rat kidney cells. *Research Communications in Molecular Pathology and Pharmacology*.

[13] Peters UR, Hasse U, Oppliger E (1999). Aberrant FHIT mRNA transcripts are present in malignant and normal haematopoiesis, but absence of FHIT protein is restricted to leukaemia. *Oncogene*.

[14] Kok K, Naylor SL, Buys CHCM (1997). Deletions of the short arm of chromosome 3 in solid tumors and the search for suppressor genes. *Advances in Cancer Research*.

[15] Pekarsky Y, Garrison PN, Palamarchuk A (2004). Fhit is a physiological target of the protein kinase Src. *Proceedings of the National Academy of Sciences of the United States of America*.

[16] Ohta M, Inoue H, Cotticelli MG (1996). The FHIT gene, spanning the chromosome 3p14.2 fragile site and renal carcinoma-associated t(3;8) breakpoint, is abnormal in digestive tract cancers. *Cell*.

[17] Huebner K, Croce CM (2003). Cancer and the FRA3B/FHIT fragile locus: it’s a HIT. *British Journal of Cancer*.

[18] Lee SH, Kim CJ, Park HK (2001). Characterization of aberrant FHIT transcripts in gastric adenocarcinomas. *Experimental and Molecular Medicine*.

[19] Holschneider CH, Baldwin RL, Tumber K, Aoyama C, Karlan BY (2005). The fragile histidine triad gene: a molecular link between cigarette smoking and cervical cancer. *Clinical Cancer Research*.

[20] Cao J, Li W, Xie J (2006). Down-regulation of FHIT inhibits apoptosis of colorectal cancer: mechanism and clinical implication. *Surgical Oncology*.

[21] Leal MF, Lima EM, Silva PNO (2007). Promoter hypermethylation od CDH1, FHIT, MTAP AND PLAGL1 in gastric adenocarcinoma in individuals from Northern Brazil. *World Journal of Gastroenterology*.

[22] Corbin S, Neilly ME, Espinosa R, Davis EM, McKeithan TW, Le Beau MM (2002). Identification of unstable sequences within the common fragile site at 3p14.2: implications for the mechanism of deletions within fragile histidine triad gene/common fragile site at 3p14.2 in tumors. *Cancer Research*.

[23] Huebner K, Hadaczek P, Siprashvili Z, Druck T, Croce CM (1997). The FHIT gene, a multiple tumor suppressor gene encompassing the carcinogen sensitive chromosome fragile site, FRA3B. *Biochimica et Biophysica Acta*.

[24] Roz L, Andriani F, Ferreira CG, Giaccone G, Sozzi G (2004). The apoptotic pathway triggered by the Fhit protein in lung cancer cell lines is not affected by Bcl-2 or Bcl-x(L) overexpression. *Oncogene*.

[25] Choi CH, Lee KM, Choi JJ (2007). Hypermethylation and loss of heterozygosity of tumor suppressor genes on chromosome 3p in cervical cancer. *Cancer Letters*.

[26] Syeed N, Husain SA, Sameer AS, Chowdhri NA, Siddiqi MA (2011). Mutational and promoter hypermethylation status of FHIT gene in breast cancer patients of Kashmir. *Mutation Research*.

[27] Jing F, Yuping W, Yong C (2010). CpG island methylator phenotype of multigene in serum of sporadic breast carcinoma. *Tumor Biology*.

[28] Ismail HMS, Medhat AM, Karim AM, Zakhary NI (2011). FHIT gene and flanking region on chromosome 3p are subjected to extensive allelic loss in egyptian breast cancer patients. *Molecular Carcinogenesis*.

[29] Benson JR (2003). The TNM staging system and breast cancer. *Lancet Oncology*.

[30] Pekarsky Y, Palamarchuk A, Huebner K, Croce CM (2002). FHIT as tumor suppressor: mechanisms and therapeutic opportunities. *Cancer Biology &amp; Therapy*.

[31] Wali A (2010). FHIT: doubts are clear now. *The Scientific World Journal*.

[32] Man S, Ellis IO, Sibbering M, Blamey RW, Brook JD (1996). High levels of allele loss at the FHIT and ATM genes in non-comedo ductal carcinoma in situ and grade I tubular invasive breast cancers. *Cancer Research*.

[33] Huiping C, Jonasson JG, Agnarsson BA, Sigbjornsdottir BI, Huebner K, Ingvarsson S (2000). Analysis of the fragile histidine triad (FHIT) gene in lobular breast cancer. *European Journal of Cancer*.

[34] Druck T, Hadaczek P, Fu TB (1997). Structure and expression of the human FHIT gene in normal and tumor cells. *Cancer Research*.

[35] Huebner K, Druck T, Siprashvili Z, Croce CM, Kovatich A, McCue PA (1998). The role of deletions at the FRA3B/FHIT locus in carcinogenesis. *Recent Results in Cancer Research*.

[36] Hayashi SI, Tanimoto K, Hajiro-Nakanishi K (1997). Abnormal FHIT transcripts in human breast carcinomas: a clinicopathological and epidemiological analysis of 61 Japanese cases. *Cancer Research*.

[37] Panagopoulos I, Thelin S, Mertens F, Mitelman F, Åman P (1997). Variable FHIT transcripts in non-neoplastic tissues. *Genes Chromosomes and Cancer*.

[38] Ahmadian M, Wistuba II, Fong KM (1997). Analysis of the FHIT gene and FRA3B region in sporadic breast cancer, preneoplastic lesions, and familial breast cancer probands. *Cancer Research*.

[39] Yang Q, Yoshimura G, Sakurai T, Kakudo K (2002). The Fragile Histidine Triad gene and breast cancer. *Medical Science Monitor*.

